# Two cases of non-intervention-related vascular intense spasm following stent implantation in the coronary artery

**DOI:** 10.3892/etm.2013.1027

**Published:** 2013-03-26

**Authors:** RIXIN XU, CHENG CHENG, FENGPU HE, XIAODONG LIU, QINGCHI LIAO, JUN JI

**Affiliations:** Cardiology Department of Subei People’s Hospital Affiliated to Yangzhou University, Yangzhou, Jiangsu 225001, P.R. China

**Keywords:** vascular intense spasm, stent implantation, coronary artery

## Abstract

The clinical occurrence of non-intervention-related vascular spasm following coronary stenting is rare. In the present study, 2 cases are reported. One patient developed continuous spasms in the proximal segment of the left anterior descending (LAD) and left circumflex (LCX) arteries following LAD artery stenting. The second patient developed an intense spasm in the right coronary artery (RCA) following LAD artery stenting. Clinical course and prognosis are dangerous. The main treatment for this condition is a combination of repeated injections of nitroglycerin into the coronary artery and the administration of calcium antagonists. In the clinic, intervention-related vascular spasms are common in percutaneous coronary intervention (PCI) due to the mechanical stimulation caused by balloon dilatation or stent expansion. Injections of a vasodilator into the coronary artery are able to mitigate the spasms and the consequent prognosis is good.

## Introduction

In the clinic, intervention-related vascular spasms are common in percutaneous coronary intervention (PCI) due to the mechanical stimulation caused by balloon dilatation or stent release (1). Injections of a vasodilator into the coronary artery are able to mitigate these spasms and consequently, the prognosis is good. However, clinical occurrences of non-intervention-related vascular spasms following coronary stenting are rare. It is also unclear as to whether the clinical manifestation and pathogenesis of non-intervention-related vascular spasms are different from those of intervention-related vascular spasms. In the present study, the clinical features and medical treatments of 2 cases of non-intervention-related vascular spasms are reported and the potential causes are discussed. Informed consent was ontained from the patient or family members.

## Case reports

### Case 1

**Figure 1:**
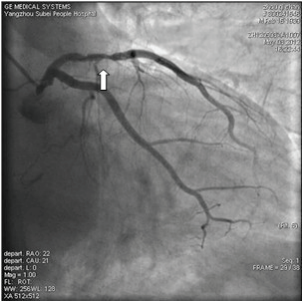
Coronary angiogram of case 1 showing that 70–80% of the left anterior descending artery stent was stenotic.

**Figure 2:**
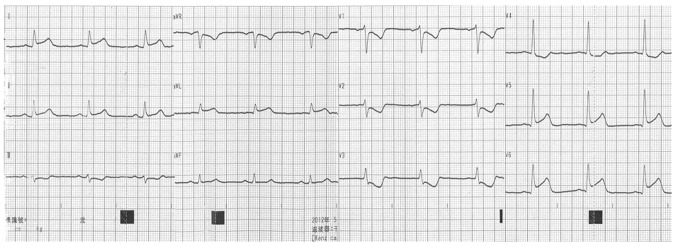
Echocardiogram of case 1 showing a significant depression in the V1-4 ST segment, inversion of the T wave and an elevation of the I, AVL and V6 ST segments.

**Figure 3:**
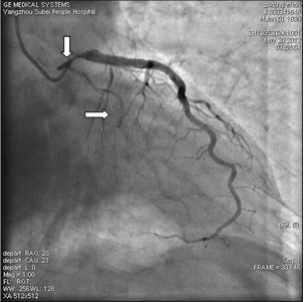
Coronary angiogram of case 1 showing severe spasms in the proximal segment of the left anterior descending artery stent and the entire left circumflex artery.

**Figure 4:**
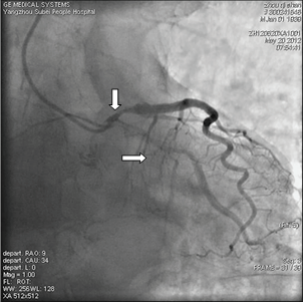
Following an intracoronary injection of nitroglycerin in case 1, the left anterior descending and left circumflex artery spasms were partially relieved.

**Figure 5:**
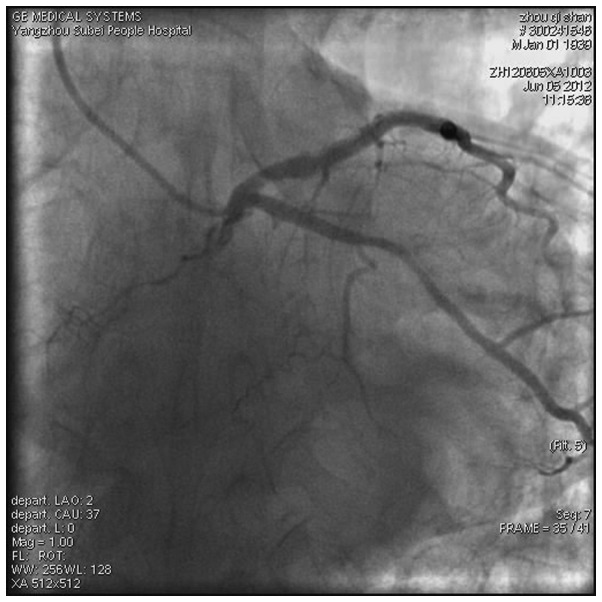
Coronary angiography review of case 1 showing that mild spasms remained in the middle of the circumflex artery.

The first patient was a 73-year-old male, whose cardiovascular risk factors included smoking for 30 years, an impaired glucose tolerance for 2 years and hypertension for >30 years. On September 9, 2009, the patient was admitted to the Subei People’s Hospital Affiliated to Yangzhou University, Jiangsu, China, due to repeated exertional chest pain. Selective coronary angiography (CAG) showed that the proximal left anterior descending (LAD) artery had a 90% segmental stenosis, while the circumflex artery and the right coronary artery (RCA) were normal. Following the implantation of a 4.0×15-mm Yinyi stent (Liaoning Biomedical Materials R&D Center Co., Ltd., Dalian, China) into the LAD lesion, there was no post-operative chest pain. On May 8, 2012, the patient was admitted again to the hospital due to unstable angina pectoris. CAG showed that the stenosis had reoccurred in 70–80% of the LAD stent (Fig. 1). The patient was hesitant whether to undergo PCI again, however on May 15, 2012, agreed to receive the treatment. The initial angiography result is shown in Fig. 1. Following pre-dilation with a 4.0×18-mm balloon at 8 atm for 10 sec, a 2.5×15-mm Resolute stent (Medtronic Inc., Galway, Republic of Ireland) was implanted in the LAD lesions. The patient immediately felt chest pain for ∼20 min following the stent implantation. The CAG review showed no abnormalities. Subsequent to the post-operative administration of a standard dual anti-platelet and statin drug, the patient did not feel the chest pain again. On May 20, 2012, on the night of the discharge day, the patient was admitted as an emergency due to sudden chest pain for 1 h with hypotension, syncope and ventricular tachycardia. On admission, echocardiography (ECG) showed significant depression in the V1-4 ST segment, inversion of the T wave and an elevation of the I, AVL and V6 ST segments (Fig. 2). Emergency CAG showed that 80–90% of the blood vessel, which is from proximal segment of the LAD stent to the LAD opening, appeared stenotic. The LAD artery stent was unobstructed. The entire left circumflex (LCX) artery appeared to be in severe spasm (Fig. 3). Following repeated injections of nitroglycerin into the coronary artery using a guiding catheter, the LAD and LCX artery spasms were partially relieved (Fig. 4). Post-operatively, a mild chest pain remained. Following the administration of a dual anti-platelet and statin and the joint use of diltiazem, nifedipine and anti-anxiety treatment for 4 days, the chest pain was totally relieved. On June 5, 2012, a mild spasm remained detectable in the middle of the circumflex artery, as shown by the CAG review (Fig. 5).

### Case 2

**Figure 6:**
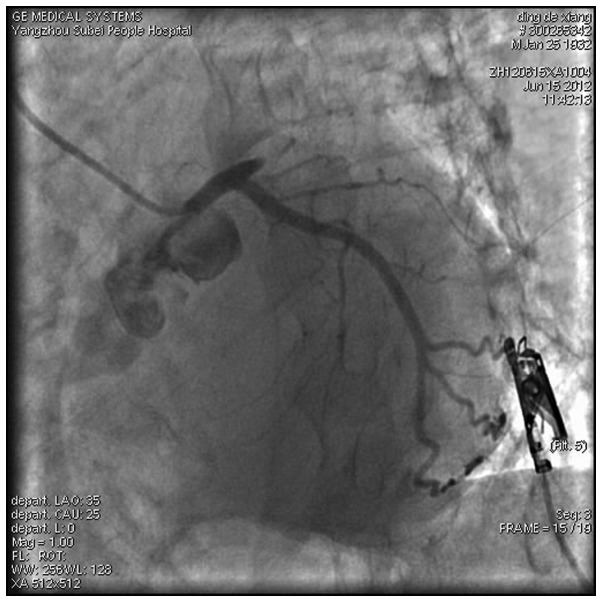
Emergency coronary angiogram of case 2 showing complete occlusion of the proximal segment of the left anterior descending artery. The left circumflex artery showed no exception.

**Figure 7:**
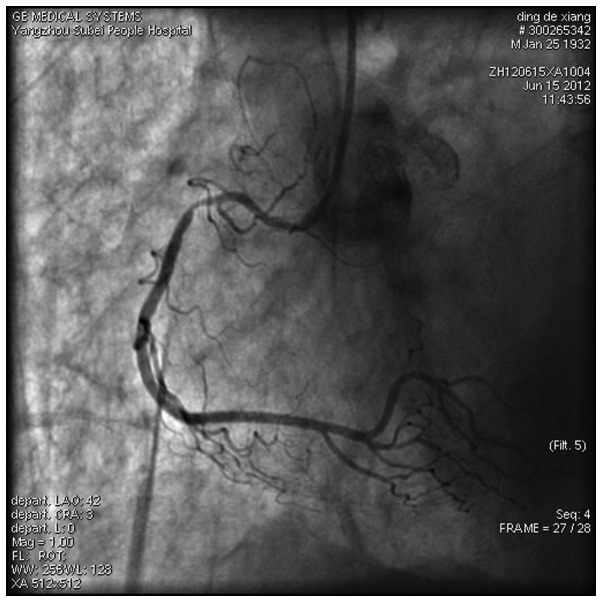
Emergency coronary angiogram of case 2 showing that 85% of the proximal segment of the right coronary artery was stenotic.

**Figure 8:**
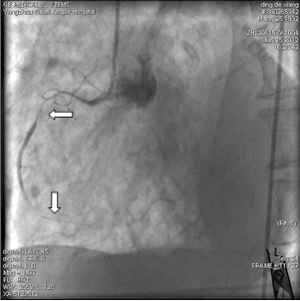
Coronary angiogram of case 2 showing intense spasms in the entire right coronary artery.

**Figure 9:**
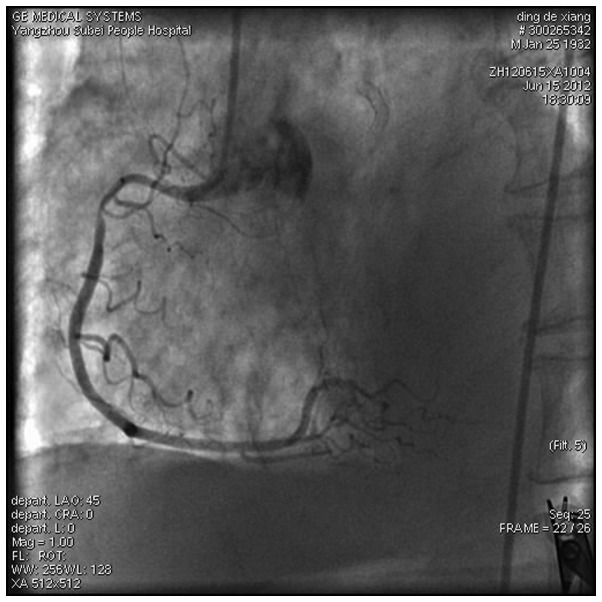
Coronary angiogram of case 2 showing that injections of nitroglycerin into the right coronary artery were able to mitigate spasms.

The second patient was an 80-year-old male, with the cardiovascular risk factors of hypertension and a history of heavy smoking. On June 15, 2012 the patient had repeated chest pain associated with a shortness of breath that had lasted for one month and was admitted to the hospital following aggravation of these symptoms for 13 h. ECG revealed acute extensive anterior myocardial infarction with cardiac dysfunction, a decrease in the motion of the anterior segmental chamber wall and a small amount of pericardial effusion. Emergency CAG revealed complete occlusion of the proximal segment of the LAD artery. The LCX artery showed no exception. The proximal segment of the RCA was shown to be 85% stenotic (Figs. 6 and 7). Following pre-dilation with a 2.5×15-mm balloon at 8 atm for 10 sec, a 3.0×33-mm Firebird stent (Shanghai MicroPort Medical Co., Ltd., Shanghai, China) was implanted in the LAD lesions. CAG showed that the blood flow in the LAD artery was between Thrombolysis In Myocardial Infarction (TIMI) levels 2 and 3. At 4 h subsequent to the emergency PCI, the patient felt a persistent chest pain again. Upon review, the ECG results showed no significant dynamic changes when compared with the first PCI. Emergency CAG showed blood flow in the LAD artery at TIMI level 3 and a strong spasm in the entire RCA (Fig. 8). Following repeated injections of nitroglycerin in the RCA using a guiding catheter, the coronary spasms were relieved (Fig. 9). During the surgery, the chest pains were also relieved. The patient did not adhere to the use of calcium antagonists following the surgery and suffered a sudden drop in blood pressure with body sweats and a slow heart rate, which showed a ventricular escape rhythm and cardiac arrest at 20 h subsequent to the second CAG. ECG revealed a pericardial effusion and cardiac rupture.

## Discussion

Angina caused by a coronary artery spasm is called vasospastic angina (VSA) (2). The usual presentation of this is temporary chest pain at rest (3). Certain cases may also present with acute myocardial infarction, malignant arrhythmias, sudden mortality and even cardiac rupture (4,5).

It is common for vascular spasms to occur due to mechanical stimulation in a remote segment of a coronary artery stent during implantation (1). An intracoronary injection of nitroglycerin is always able to quickly alleviate the spasms. There have also been studies reporting spontaneous spasms at the stent edges following several months of use (6). The prognosis of these cases is usually good. There are also rare clinical occurrences of non-intervention-related vascular spasm following coronary stenting. Versaci *et al* reported one case with LAD and LCX artery spasms following RCA stent implantation (7). In this report, the coronary spasm was transient and did not cause serious consequences. Wong *et al* reported one case with a sustained, serious and widespread spasm in the entire left coronary system during LAD coronary artery stenting. A coronary injection of vasodilator using a guiding catheter was unsuccessful in this patient who suffered acute pulmonary edema and cardiogenic shock. The coronary spasm was finally relieved by an injection of nitroglycerin into the distal segment of the spastic vessel through a micro-catheter (8).

The present study reported two cases of patients with the following characteristics: i) The non-invasive vascular system was involved in the coronary artery spasms. In case 1 the left coronary system showed widespread spasms, involving the LAD and LCX arteries following LAD artery stenting. In case 2, the RCA showed spasms at all stages following LAD artery stenting. ii) Strong spasms caused coronary artery lumen occlusion. The spasms were long lasting, stubborn and only partially relieved following multiple injections of nitroglycerin through the coronary artery; furthermore, repeated attacks were observed within a short time. In case 1, four days of post-operative joint administration of diltiazem, nifedipine and anti-anxiety treatment were required to fully control the chest pain. It was unfortunate that the patient from case 2 did not adhere to the application of calcium antagonists to prevent the cramps that recurred following reduction of the spasms. iii) The risk of clinical symptoms: There were widespread spasms in the LAD and LCX coronary arteries of case 1, similar to a blockage in the left main coronary artery, resulting in hypotension, syncope and ventricular tachycardia. The patient in case 2 had persistent chest pain following LAD artery emergency stenting. Emergency CAG showed that the blood flow in the LAD artery was significantly improved but that a strong spasm remained in the RCA. The patient subsequently succumbed to a cardiac rupture. It is not possible to rule out the strong spasms in the RCA as a factor in the patient’s mortality.

Coronary spasms are associated with a variety of factors, including strenuous exercise, excessive expansion of the coronary artery, gene polymorphisms of endothelial nitric oxide synthase, hyperthyroidism, smoking, the use of dobutamine (9) and cocaine, and drinking. Stent implantation may aggravate or cause endothelial dysfunction. Vasoconstriction is enhanced at each end of the stent. The implantation of a drug-eluting stent is more likely to induce a vascular spasm (10,11). Kim *et al* reported a case with acute inferior myocardial infarction. Drug-eluting stents were implanted into the RCA and LAD and LCX arteries, and 10 h later the wide spasms appeared in the left coronary artery and RCA. The main cause of the multiple vasospasm induction was proposed to be an endothelial injury during the process of drug stent implantation (12). In the present study, the patient in case 1 felt chest pain for ∼20 min immediately after stent implantation in the LAD coronary artery. However, the CAG results were normal. The strong coronary spasm on the fourth day subsequent to stenting may have been related to the selection of a larger stent and the stimulation of excessive traction in the stenting segment of the LAD artery. Also, the patient demonstrated an evident anxiety prior to the stent implantation, therefore, when the review CAG showed in-stent restenosis, the patient did not agree to receive the same level of PCI. We believe that emotional anxiety in patients may be a factor that aggravates coronary spasms. The patient in case 2 had an acute occlusion of the LAD artery. The reason for the spasm in the RCA following stenting in the LAD artery remains unclear. However, the reason may be that the body was in a state of stress due to the release of catecholamines and inflammatory mediators as a consequence of the acute myocardial infarction.

In summary, there are rare clinical occurrences of non-intervention-related vascular spasms following coronary stenting. These spasms have the characteristics of a broad, strong and long duration with repeated episodes. The clinical course has dangers. In emergencies, repeated injections of vasodilator into the coronary artery and rapid remission of the spasms are important for salvage and therapy. Post-operative high-dose calcium antagonists and a mechanism-focused treatment for the spasms may also be extremely important.

## References

[1] Fischell TA (1990). Coronary artery spasm after percutaneous transluminal coronary angioplasty: pathophysiology and clinical consequences. Cathet Cardiovasc Diagn.

[2] Kusama Y, Kodani E, Nakagomi A (2011). Variant angina and coronary artery spasm: the clinical spectrum, pathophysiology, and management. J Nippon Med Sch.

[3] Prinzmetal M, Kennamer R, Merliss R (1959). Angina pectoris. I. A variant form of angina pectoris; preliminary report. Am J Med.

[4] Hillis LD, Braunwald E (1978). Coronary-artery spasm. N Engl J Med.

[5] Maseri A, L’Abbate A, Baroldi G (1987). Coronary vasospasm as a possible cause of myocardial infarction. A conclusion derived from the study of ‘preinfarction’ angina. N Engl J Med.

[6] Kaku B, Kanaya H, Horita Y (2005). Spontaneous stent-edge spasm in a patient with myocardial infarction. Heart.

[7] Versaci F, Gaspardone A, Proietti I (2002). Left anterior descending and circumflex coronary artery spasm after right coronary artery stent implantation. Heart.

[8] Wong A, Cheng A, Chan C (2005). Cardiogenic shock caused by severe coronary artery spasm immediately after coronary stenting. Tex Heart Inst J.

[9] Bonvini RF, Hendiri T, Sigwart U (2006). Angiographic documented dobutamine induced coronary spasm successfully treated by stenting. Heart.

[10] Kitahara H, Kobayashi Y, Iwata Y (2011). Effect of pioglitazone on endothelial dysfunction after sirolimus-eluting stent implantation. Am J Cardiol.

[11] Ito S, Nakasuka K, Morimoto K (2011). Angiographic and clinical characteristics of patients with acetylcholine-induced coronary vasospasm on follow-up coronary angiography following drug-eluting stent implantation. J Invasive Cardiol.

[12] Kim JW, Park CG, Seo HS, Oh DJ (2005). Delayed severe multivessel spasm and aborted sudden death after Taxus stent implantation. Heart.

